# Depression, anxiety and stress symptoms among diabetics in Malaysia: a cross sectional study in an urban primary care setting

**DOI:** 10.1186/1471-2296-14-69

**Published:** 2013-05-27

**Authors:** Gurpreet Kaur, Guat Hiong Tee, Suthahar Ariaratnam, Ambigga S Krishnapillai, Karuthan China

**Affiliations:** 1 Institute for Public Health, Ministry of Health, Jalan Bangsar, Kuala Lumpur, 50590, Malaysia; 2 Faculty of Medicine, Universiti Teknologi MARA, Selayang Campus, Jalan Prima Selayang 7, Batu Caves, Selangor, 68100, Malaysia; 3 Department of Social and Preventive Medicine, University of Malaya, Kuala Lumpur, 50603, Malaysia

**Keywords:** Depression, Anxiety, Stress, Prevalence, Predictors, Diabetes, Outpatients, Urban, Malaysia

## Abstract

**Background:**

Diabetes mellitus is a highly prevalent condition in Malaysia, increasing from 11.6% in 2006 to 15.2% in 2011 among individuals 18 years and above. Co-morbid depression in diabetics is associated with hyperglycemia, diabetic complications and increased health care costs. The aims of this study are to determine the prevalence and predictors of depression, anxiety and stress symptoms in Type II diabetics attending government primary care facilities in the urban area of Klang Valley, Malaysia.

**Methods:**

The study was cross sectional in design and carried out in 12 randomly selected primary care government clinics in the Klang Valley, Malaysia. A total of 2508 eligible consenting respondents participated in the study. The Depression, Anxiety and Stress Scale (DASS) 21 questionnaire was used to measure depression, anxiety and stress symptoms. Data was analyzed using the SPSS version 16 software using both descriptive and inferential statistics.

**Results:**

The prevalence of depression, anxiety and stress symptoms among Type II diabetics were 11.5%, 30.5% and 12.5% respectively. Using multiple logistic regression, females, Asian Indians, marital status (never married, divorced/widowed/separated), a family history of psychiatric illness, less than 2 years duration of diabetes and current alcohol consumption were found to be significant predictors of depression. For anxiety, unemployment, housewives, HbA1c level of more than 8.5%, a family history of psychiatric illness, life events and lack of physical activity were independent risk factors. Stress was significantly associated with females, HbA1c level of more than 8.5%, presence of co-morbidity, a family history of psychiatric illness, life events and current alcohol consumption. For depression (adjusted OR 2.8, 95% CI 1.1; 7.0), anxiety (adjusted OR 2.4, 95% CI 1.1;5.5) and stress (adjusted OR 4.2, 95% CI 1.8; 9.8), a family history of psychiatric illness was the strongest predictor.

**Conclusion:**

We found the prevalence of depression, anxiety and stress symptoms to be high among Type II diabetics, with almost a third being classified as anxious. Screening of high risk Type II diabetics for depression, anxiety and stress symptoms in the primary care setting is recommended at regular intervals.

## Background

Diabetes and depression are two of the commonest public health problems affecting people all over the world. About 220 million people are estimated to be suffering from diabetes, majority of the burden being in low and middle income countries (LMIC) [1]. Diabetes is also responsible for about 1.256 million deaths globally in 2008, with most deaths occurring in LMIC. Unipolar depressive disorders and diabetes were ranked 3^rd^ and 19^th^ respectively as leading causes of disability adjusted life years (DALYs) in 2004. The former also being the leading cause of years lost due to disability (YLD). Unipolar depressive disorders are in fact projected to be the leading cause of disease burden by 2030.

In Malaysia, a middle income country, the prevalence of diabetes has increased in the last decade from 5.7% to 9.5% among individuals aged 30 years and above [2] and from 11.6% in 2006 to 15.2% in 2011 among individuals 18 years and older[3]. Of the estimated 2.6 million diabetics in Malaysia, about 715,550 (27.5%) diabetics are originating from the most populous regions in Malaysia namely the state of Selangor and the Federal Territory of Kuala Lumpur [3]. In terms of leading causes of total YLD, the Malaysian National Burden of Disease and Injury Study 2004 ranked diabetes mellitus as the third leading cause in both males (6.0%) and females (7.2%) respectively, while unipolar major depression was ranked as second and top most leading cause in males (7.2%) and females (12.7%) respectively [4].

It is well recognized that many individuals with chronic illnesses also have co-morbid unrecognized mental health disorders [5]. The International Federation of Diabetes has stressed the importance of integrating psychological care in the management of diabetes [6].

It has been estimated that the risk of getting depression in the general population is 10-25% in females and 5 – 12% in males. For individuals with chronic illnesses, the risk is higher at 25 – 33% [7]. Studies have shown that diabetics have a higher prevalence of depression than non-diabetic populations [8-10]. Globally, an estimated 43 million diabetics have symptoms of depression [5]. Also, diabetes is associated with anxiety disorders [11]. Being diagnosed with diabetes is a life stressor by itself. It requires a large number of physical and mental accommodations. Depression adds to the burden of managing diabetes. Furthermore, health care utilization and costs [12-14] increase with the coexistence of diabetes and major depression.

Depression and anxiety are associated with hyperglycemia [15-17]. While depression is associated with diabetes complications [18,19] and increased functional disability [20,21]. Co-morbid depression has also been shown to be associated with poor adherence to diabetes medication and dietary regimens [16,22,23] and reduced quality of life [24,25]. Several studies have shown the risk of mortality to be increased by depression [26-29]. The PROSPECT trial has shown that the five year risk of mortality was reduced with a depression management care program among diabetics compared to similar patients with usual care practices [30].

In Malaysia, there is paucity of epidemiological estimates on the prevalence, characterization and risk factors of depression, anxiety and stress among diabetics. An estimate of the prevalence of these conditions is the first step towards priority setting and the planning, implementation and evaluation of a depression management intervention program in diabetes care in the primary care setting. This study was carried out with the aims of determining the prevalence of depression, anxiety and stress, and its predictors among Type II diabetic outpatients attending government primary care clinics in the Klang Valley.

## Methods

This was a cross sectional study carried out in 12 selected government primary care clinics located in the Klang Valley.

### Study location

The Klang Valley generally refers to the urban areas of Kuala Lumpur, its suburbs and adjoining areas in the state of Selangor. To the North and East, it is demarcated by the Titiwangsa Mountain range, while to the West by the Straits of Malacca. The estimated population of the Klang Valley is 7.5 million [31]. The two federal territories of Kuala Lumpur and Putrajaya as well as five districts from the state of Selangor (Sepang, Hulu Langat, Gombak, Klang and Petaling) were included in the study. In each of these localities the number of clinics ranged from 1 to 13 with a total of 45 clinics.

### Sample size and sampling

Sample size was calculated using both the population survey method for prevalence and for comparing two proportions using the Sample Size Calculator for Prevalence Studies [32] and the PS Software [33] respectively. The larger minimum sample size required based on both these methods was taken as the sample size for the whole study. Based on 80% study power, Type I error of 0.05, design effect of 2, a difference of 8% in two groups and a non-response of 20%, a sample size of 2261 was required.

For sampling, a two stage stratified sampling technique was employed. About 25% of the total number of clinics from each locality (with a minimum of one clinic) was randomly selected, giving a total of 12 clinics from the study area. The sample size was then proportionately distributed based on the number of clinics selected from each locality. For three localities where only one single clinic was selected, the minimum sample size was increased to at least 250 to enable clinic level analyses in future. The final minimum sample size for the study was 2446.

### Study procedure

All patients attending the diabetic clinics during the study period were screened for eligibility to participate. The inclusion criteria were age 30 years and above, having Type II diabetes of at least six months (verified with medical records) and being literate in Malay (which is the official language of the country) or English. Patients with a known medically diagnosed psychiatric illness in the past (verified with medical records) or with any form of cognitive impairment such as dementia or mental retardation and females in the post-partum period were excluded. Eligible patients were then approached for written consent for the study. Prior to obtaining consent, all potential respondents were explained about the purpose of the study and the relevant procedures involved. They were assured that their blood results would be notified eventually to their attending physician. Subsequently, only consenting patients were recruited in the study.

### Ethical issues

Ethical approval for the study was obtained from the Medical Research Ethics Committee, Ministry of Health Malaysia (NIHSEC 08/0809/P09). Permission to conduct the study was also obtained from the State Health Directors as well as Medical and Health Officers in charge of the selected clinics prior to the study.

### Data collection tools and measurements

Socio-demographic and other relevant information were collected by five trained interviewers via face-to-face interview. Depression, anxiety and stress symptoms were measured using a self-administered short version of the Depression, Anxiety and Stress Scale (DASS), i.e., DASS 21 (Additional file 1: DAS S 21). The short version has 21 items which are divided into 7 items each assessing the symptoms of depression, anxiety and stress respectively. The DASS has been shown to have high internal consistency. The validated Malay or Bahasa Malaysia version of DASS 21 was used in this study [34]. Respondents were asked to rate their experience on each symptom over the past week on a 4-point severity scale ranging from 0 (does not apply to me), to 3 (applies to me most or all of the time). Scores for each scale were later summed up and categorized as normal, mild, moderate, severe and extremely severe according to the DASS Manual [35].

The following anthropometric and blood assay measurements were also taken:

Height and weight for Body Mass Index (BMI) - Both parameters were measured twice with the patient standing bare footed. Height (to the nearest centimeter) was measured with fixed stadiometers (Seca, Vogel & Halke, Germany) and weight (to the nearest 0.1 kilogram) was measured using an electronic floor weighing scale (Tanita HD 319 Personal Scale, Australia). BMI was classified according to the World Health Organisation guidelines [36].

Waist circumference (WC) - Measured twice using a standard tape measure as described by the National Institutes of Health (NIDDK) [37]. Abdominal obesity was defined by a waist circumference of ≥90 cm for men and ≥80cm for women [38].

Total Serum Cholesterol (TC) - Measured using the Accutrend GCT (Roche Diagnostics, Germany) from a single finger prick.

HbA1c level - Measured using the DCA Vantage Analyser (Siemens Healthcare Diagnostics Inc, USA) from the same single finger prick.

For all the measurements except TC and HbA1c an average of two readings was taken for analyses. Variables were categories based on clinical and statistical reasoning.

### Pre-test and pilot study

A pre-test was conducted on 40 Type II diabetic patients selected conveniently from a government primary care facility which was not included in the study. This was purposively done to test the study questionnaire. The pilot study was then conducted after the pre-test in two other government health facilities that were also not selected for the study. The logistics and feasibility of conducting the study were explored. Weaknesses that were identified from the pre-test and pilot study were rectified.

### Data management and statistical analysis

All questionnaires were checked for completeness of response at the clinic and attempts were made to improve the response rate for missing items. Data was entered manually into a database and cleaned before analyses. The Statistical Package for the Social Science (SPSS) version 16 software was used for both descriptive and inferential analysis. Items that were not answered by respondents were considered as missing. Univariate statistics such as mean values, standard deviations, frequencies and proportion percentages were derived for continuous and categorical variables respectively. Bivariate and multivariate analyses were used to measure the strength of association between the variables in the study and identify predictors for the outcomes of interest respectively. All tests were two-tailed with significance defined as p < 0.05. Odds ratios (OR) along with 95% confidence levels (CI) were derived where appropriate.

## Results

Out of 2774 eligible patients approached, 2508 subjects were successfully recruited, giving a response rate of 90.4%.

### Socio-demographic, clinical and other characteristics of sample population

Majority of the respondents were between 50 to 59 years old (35.5%), females (61.1%), Malays (51.2%) and married (81.7%). Almost 40% had some form of secondary education and one third earned a monthly household income (MHI) of MYR 1,000 to 1,999. Slightly over one third of respondents were gainfully employed. The mean age and MHI was about 57 (56.6 ± SD 10.67) years and MYR 2,000 (1,974.6 ± SD 1,869.12) respectively (Table 1).

**Table 1:** Frequency distribution of respondents by socio-demographic characteristics and selected variables

**Demographic characteristics (n=2508)**	**Mean**	**SD**	**n**	**Percentage (%)**
**Age (years)**	56.6	10.67		
30 – 39			158	6.3
40 – 49			458	18.3
50 – 59			891	35.5
60 – 69			728	29.0
70 – 79			238	9.5
≥ 80			35	1.4
**Sex**				
Male			975	38.9
Female			1533	61.1
**Ethnicity**^**a**^				
Malay			1282	51.2
Chinese			438	17.5
Asian Indian			787	31.3
**Highest Educational Level**				
None			285	11.4
Never completed primary school			423	16.9
Completed primary school			519	20.7
Never completed secondary school			462	18.4
Completed secondary school			523	20.9
A-Level/STPM/HSC			184	7.3
Tertiary			112	4.5
**Monthly household income (MHI) in Malaysian Ringgit (MYR**^**b**^**)**	1,974.6	1,869.12		
< 400			114	4.5
400 – 699			277	11.0
700 – 999			313	12.5
1000 – 1999			798	31.8
2000 – 2999			510	20.3
3000 – 3999			207	8.3
4000 – 4999			105	4.2
≥ 5000			184	7.3
**Marital Status**				
Never married			71	2.8
Married/Cohabiting			2048	81.7
Divorced/Separated/Widowed			389	15.5
**Current Job Status**				
Civil servant			227	9.0
Private Sector employee			448	17.8
Self-employed			171	6.8
Government retiree			210	8.4
Private retiree			158	6.3
Studying and working			2	0.2
Student			2	0.1
Housewife			781	31.1
Unemployed			509	20.3
**BMI (kg/m**^**2**^**)**	27.83	5.395		
Underweight (< 18.5)			28	1.1
Normal (≥ 18.5 - < 25.0)			751	29.9
Overweight (≥ 25.0 - < 30.0)			1034	41.2
Obese (≥ 30.0)			695	27.7
**Abdominal Obesity**				
No			540	21.5
Yes			1968	78.5
**Serum Cholesterol (mmol/l)**	5.03	0.878		
< 5.2			1767	70.5
≥ 5.2			741	29.5
**HbA1c (%)**^**a**^	8.36	2.038		
≤8.5			1537	61.3
>8.5			970	38.7
**Duration of Diabetes (years)**	7.7	6.26		
< 2 yrs			206	8.2
2 - < 10 yrs			1521	60.6
10 - < 20 yrs			586	23.4
≥ 20 yrs			195	7.8
**Co-morbidity**^**c**^				
No			530	21.1
Yes			1978	78.9
**Psychiatric illness in family**				
No			2484	99.0
Yes			24	1.0
**Diabetes in family**				
No			661	26.4
Yes			1847	73.6
**Life events within the last 6 months**				
No			1373	54.7
Yes			1135	45.3
**Smoking status**^**†**^				
Non-smoker			2089	83.3
Former smoker			160	6.4
Current smoker			259	10.3
**Alcohol Consumption Status‡**				
Lifetime abstainer			2326	92.7
Former drinker			88	3.5
Current drinker			94	3.8
**Leisure-time Physical Activity Status§**				
Regular activity			793	31.6
Some activity			483	19.3
Inactive			1232	49.1
**Life events within the last 6 months**¶				
No			1373	54.7
Yes			1135	45.3

^a^ one missing data.

^b^1 USD is approximately 3 MYR.

^c^ Co-morbidity –any chronic co-morbid condition present.

^†^ Non-smoker - Respondent who reported to have never smoked at least 100 cigarettes in his lifetime.

Current smoker- Respondents who reported to have smoked 100 or more cigarettes in his lifetime and currently smoked daily or some days.

Former smoker - Respondents who reported to have smoked 100 or more cigarettes in his lifetime but not smoking currently.

‡ a) Current drinker – persons who have had at least 12 drinks in their lifetime and at least one drink in the previous year.

b) Former drinker - persons who have had ≥12 drinks in their lifetime, but no drinks in the past year.

c) Lifetime abstainer – had < 12 drinks in his/her entire lifetime.

§a) Inactive – did not report any sessions of light to moderate or vigorous leisure-time physical activity of at least 10 minutes or reported they were unable to perform leisure-time physical activity.

b) Some leisure-time activity – at least 1 session of light to moderate or vigorous activity of at least 10 minutes in duration but did not meet the requirement of regular leisure-time activity.

c) Regular leisure-time activity – at least 3 sessions per week of vigorous leisure-time activity lasting at least 20 minutes or at least 5 sessions per week of light to moderate physical activity lasting at least 30 minutes or both.

¶ Life events – any sudden change in one’s life whether desirable or undesirable.

Over 41% of the respondents were overweight while almost twice the proportion had abdominal obesity. Almost 30% and 40% of the respondents had an elevated TC level of >5.2 mmol and HbA1c of >8.5% respectively. Majority (61%) were diagnosed as having diabetes for 2 - <10 years (Table 1).

Almost 80% reported having at least one co-morbid condition. Majority reported having at least one family member with diabetes (73.6%) while only 1% reported having a history of mental illness in the family. As for life events, about 45% reported experiencing at least one life event in the past 6 months. Current smokers and current alcohol drinkers comprised 10% and 4% of the respondents respectively. Almost half of the respondents were classified as physically inactive (49.1%).

### Depression, anxiety and stress

Overall, the prevalence of depression, anxiety and stress symptoms were 11.5%, 30.5% and 12.5% respectively. On bivariate analysis using binary logistic regression, depression was found to be significantly associated with sex, ethnicity, educational level, MHI, marital status, current job status, duration of diabetes and family history of psychiatric illness (Table 2).

**Table 2:** Frequency distribution of respondents by depression status and socio-demographic characteristics and other selected variables

**Variables**	**Depression symptoms**				***P***	_**c**_**OR**	**95% CI**	
	**No (n) **^**†**^	**(%)**	**Yes (n) ‡**	**(%)**			**Lower**	**Upper**
**Age (Years)**					0.593			
30 – 39*	138	87.3	20	12.7	-	-	-	-
40 - 49	407	88.3	54	11.7	0.752	0.915	0.53	1.58
50 - 59	796	89.5	93	10.5	0.413	0.806	0.48	1.35
60 – 69	645	88.6	83	11.4	0.655	0.888	0.53	1.50
70 - 79	205	86.9	33	13.1	0.730	1.111	0.61	2.02
≥ 80	28	82.4	6	17.6	0.443	1.479	0.55	4.01
**Sex**								
Male*	886	90.9	89	9.1	-	-	-	-
Female	1332	87.0	200	13.0	0.030	1.49	1.15	1.94
**Ethnicity (n=2507)**					0.006			
Malay*	1155	90.1	127	9.9		-	-	-
Chinese	390	89.0	48	11.0	0.529	1.12	0.79	1.59
Asian Indian	673	85.5	114	14.5	0.002	1.54	1.18	2.02
**Educational Level**					0.006			
None	231	81.9	54	18.1	0.001	2.24	1.37	3.65
Primary education	837	88.9	105	11.1	0.414	1.20	0.77	1.86
Secondary education	883	89.6	102	10.4	0.655	1.11	0.71	1.72
Tertiary education*	268	90.5	28	9.5	-	-	-	-
**Monthly household income (MHI) in Malaysian Ringgit (MYR)**					0.006			
< 1,000	92	80.7	22	19.3	0.001	1.90	1.28	2.80
1,000 -< 3,000	238	85.9	39	14.1	0.021	1.54	1.07	2.23
≥ 3,000*	276	88.2	37	11.8	-	-	-	-
**Marital Status**					0.000			
Never married	55	77.5	16	22.5	0.001	2.72	1.53	4.83
Divorced/widowed/separated	1850	90.3	198	9.7	0.000	2.23	1.67	2.99
Married*	314	80.7	75	19.3	-	-	-	-
**Current Job Status**					0.013			
Unemployed	438	85.4	75	14.6	0.005	1.62	1.16	2.26
Housewives	682	87.3	99	12.7	0.047	1.37	1.00	1.82
Retired	334	90.8	34	9.2	0.855	0.96	0.63	1.46
Employed*	765	90.4	81	9.6	-	-	-	-
**BMI (kg/m**^**2**^**)**					0.207			
< 25.0*	686	88.1	93	11.9	-	-	-	-
≥ 25.0	928	89.7	106	10.3	0.256	0.84	0.63	01.13
≥30.0	605	87.1	90	12.9	0.557	1.10	0.81	1.50
**Abdominal Obesity**								
No *	484	89.6	56	10.4	-	-	-	-
Yes	1735	88.2	233	11.8	0.344	1.16	0.85	1.58
**Sr. Cholesterol level (mmol/l)**								
<5.2*	1555	88.0	212	12.0	-	-	-	-
≥ 5.2	664	89.6	77	10.4	0.251	0.85	0.65	1.12
HbA1c level (%)								
≤8.5 *	1369	89.1	168	10.9	-	-	-	-
>8.5	849	87.5	121	12.5	0.239	1.16	0.91	1.49
**Duration of diabetes (years)**								
< 2	173	84.0	33	16.0	0.036	1.53	1.03	2.26
≥ 2*	2046	88.9	256	11.1	-	-	-	-
**Co-morbidity**								
No*	466	87.9	64	12.1	-	-	-	-
Yes	1753	88.6	225	11.4	0.654	0.94	0.70	1.26
**Psychiatric illness in family**								
No*	2202	88.6	282	11.4	-	-	-	-
Yes	17	70.8	7	29.2	0.010	3.22	1.32	7.82
**Diabetes in family**								
No*	582	88.0	79	12.0	-	-	-	-
Yes	1637	88.6	210	11.4	0.688	0.945	0.718	1.245
**Life events within the past 6 months**								
No*	1221	88.9	152	11.1	-	-	-	-
Yes	998	87.9	137	12.1	0.435	1.103	0.863	1.410
**Smoking status**					0.550			
Non smoker*	1842	88.2	247	11.8	-	-	-	-
Former smoker	143	89.4	17	10.6	0.304	0.797	0.517	1.229
Current smoker	234	90.3	25	9.7	0.650	0.887	0.527	1.491
**Current drinker**								
No*	2140	88.6	274	11.4	-	-	-	-
Yes	79	84.0	15	16.0	0.173	1.48	0.84	2.61
**Leisure-time physical activity level**								
Inactive	1077	87.4	155	12.6	0.103	1.23	0.96	1.57
Active*	1142	89.5	134	10.5	-	-	-	-

_c_OR –Crude odds ratio.

*- Reference group.

^†^ - Scores of 0–9 (normal).

‡ − Scores of ≥ 10 (mild, moderate, severe, extremely severe).

For anxiety, all socio-demographic variables; females, Asian Indian, no formal education, MHI of MYR < 3,000, divorcees/widowers, housewives and unemployment were significant. In addition, HbA1c >8.5%, ≥ 2 years duration of diabetes, presence of psychiatric illness in the family, life events and physical inactivity were also significantly associated (Table 3).

**Table 3:** Frequency distribution of respondents by anxiety status and socio-demographic characteristics and other selected variables

**Variables**	**Anxiety symptoms**				***P***	_**c**_**OR**	**95% CI**	
	**No (n) **^**†**^	**(%)**	**Yes (n) ‡**	**(%)**			**Lower**	**Upper**
**Age (Years)**					0.006			
30 – 39*	102	64.6	56	35.4	-	-	-	-
40 - 49	341	74.2	119	25.8	0.021	0.63	0.43	0.93
50 - 59	628	70.6	261	29.4	0.126	0.76	0.53	1.08
60 – 69	504	69.2	224	30.8	0.253	0.81	0.56	1.16
70 - 79	144	60.5	94	39.5	0.416	1.19	0.78	1.80
≥ 80	22	64.7	12	35.3	0.987	0.99	0.46	2.16
**Sex**								
Male*	710	72.8	265	27.2	-	-	-	-
Female	1032	67.3	501	32.7	0.004	1.30	1.09	1.55
**Ethnicity**					0.022			
Malay*	911	71.0	372	29.0	-	-	-	-
Chinese	314	71.7	124	28.3	0.778	0.97	0.76	1.23
Asian Indian	516	65.7	270	34.3	0.012	1.28	1.06	1.55
**Educational Level**					0.070			
None	184	64.6	101	35.4	0.045	1.43	1.01	2.04
Primary education	629	66.8	313	33.2	0.076	1.30	0.97	1.73
Secondary education	715	72.6	270	27.4	0.922	0.99	0.74	1.32
Tertiary education*	214	72.3	82	27.7	-	-	-	-
**Monthly household income (MHI) in Malaysian Ringgit (MYR)**					0.001			
< 1,000	457	64.9	247	35.1	0.000	1.64	1.27	2.12
1,000 -< 3,000	912	69.7	396	30.3	0.022	1.32	1.04	1.67
≥ 3,000*	373	75.2	123	24.8	-	-	-	-
**Marital Status**					0.010			
Never married	52	73.2	19	26.8	0.626	0.88	0.51	1.49
Divorced/widowed/separated	1445	70.6	603	29.4	0.003	1.41	1.12	1.77
Married*	245	63.0	144	37.0	-	-	-	-
**Current Job Status**					0.000			
Unemployed	315	61.4	198	38.6	0.000	1.86	1.47	2.35
Housewives	530	67.9	251	32.1	0.002	1.40	1.13	1.74
Retired	265	72.0	103	28.0	0.326	1.15	0.87	1.51
Employed*	632	74.7	214	25.3	-	-	-	-
**BMI (kg/m**^**2**^**)**					0.472			
< 25.0*	529	67.9	250	32.1	-	-	-	-
≥ 25.0	721	69.7	313	30.3	0.407	0.92	0.75	1.12
≥ 30.0	492	70.8	203	29.2	0.023	0.87	0.70	1.09
**Abdominal Obesity**								
No*	373	69.1	167	30.9	-	-	-	-
Yes	1369	69.6	599	30.4	0.827	0.98	0.80	1.20
**Sr. Cholesterol level (mmol/l)**								
<5.2*	1215	68.8	552	38.2	0.242	0.90	0.74	1.08
≥ 5.2	527	71.1	214	28.9	-	-	-	-
**HbA1c level (%)**								
≤8.5*	1101	71.6	436	28.4	-	-	-	-
>8.5	641	66.1	329	33.9	0.003	1.30	1.09	1.54
**Duration of DM (years)**								
< 2	158	76.7	48	23.3	0.019	1.49	1.07	2.09
≥ 2*	1584	68.8	718	31.2	-	-	-	-
**Co-morbidity**								
No*	382	72.1	148	27.9	-	-	-	-
Yes	1360	68.8	618	31.2	0.141	1.17	0.95	1.45
**Psychiatric illness in family**								
No*	1730	69.6	754	30.4	0.043	2.29	1.03	5.13
Yes	12	50.0	12	50.0	-	-	-	-
**Diabetes in family**								
No*	477	72.2	184	37.8	0.079	1.19	0.98	1.45
Yes	1265	68.5	582	31.5	-	-	-	-
**Life events within the past 6 months**								
No*	996	72.5	377	27.5	-	-	-	-
Yes	746	65.7	389	34.3	0.000	1.38	1.16	1.63
**Smoking status**					0.064			
Non smoker*	1433	68.6	656	31.4	-	-	-	-
Former smoker	186	71.8	73	28.2	0.030	0.66	0.45	0.96
Current smoker	123	76.9	37	23.1	0.292	0.86	0.64	1.14
**Current drinker**								
No*	1677	69.5	737	30.5	-	-	-	-
Yes	65	69.1	29	30.9	0.947	1.015	0.650	1.59
**Leisure-time physical activity level**								
Active*	931	73.0	345	27.0	-	-	-	-
Inactive	811	65.8	421	34.2	0.000	1.40	1.18	1.66

_c_OR –Crude odds ratio.

*- Reference group.

^†^ - Scores of 0–7 (normal).

‡ − Scores of ≥ 8 (mild, moderate, severe, extremely severe).

Stress was significantly associated with sex, HbA1c, co-morbidity, diabetes in the family, psychiatric illness in the family, life events and alcohol consumption (Table 4).

**Table 4:** Frequency distribution of respondents by stress status and socio-demographic characteristics and other selected variables

**Variables**	**Stress symptoms**				***P***	_**c**_**OR**	**95% CI**	
	**No (n) **^**†**^	**(%)**	**Yes (n) ‡**	**(%)**			**Lower**	**Upper**
**Age (Years)**					0.336			
30 – 39*	133	84.2	25	15.8	-	-	-	-
40 - 49	408	88.5	53	11.5	0.159	0.691	0.413	1.156
50 - 59	784	88.2	105	11.8	0.160	0.713	0.444	1.144
60 – 69	627	86.1	101	13.9	0.525	0.857	0.532	1.380
70 - 79	214	89.9	24	10.1	0.092	0.597	0.327	1.088
≥ 80	28	82.4	6	17.6	0.793	1.140	0.428	3.037
**Sex**								
Male*	870	89.2	105	10.8	-	-	-	-
Female	1324	86.4	209	13.6	0.035	1.31	1.02	1.68
**Ethnicity**					0.077			
Malay*	1135	88.5	147	11.5	-	-	-	-
Chinese	387	88.4	51	11.6	0.920	1.02	0.73	1.43
Asian Indian	671	85.3	116	14.7	0.030	1.34	1.03	1.73
**Educational Level**					0.219			
None	253	88.8	32	11.2	0.484	1.211	0.709	2.068
Primary education	824	87.5	118	12.5	0.155	1.371	0.888	2.116
Secondary education	849	86.2	136	13.8	0.051	1.533	0.998	2.355
Tertiary education*	268	90.5	28	9.5	-	-	-	-
**Monthly household income (MHI) in Malaysian Ringgit (MYR)**					0.088			
< 1,000	600	85.2	104	14.8	0.051	1.419	1.00	2.02
1,000 -< 3,000	1152	88.1	156	11.9	0.539	1.108	0.80	1.54
≥ 3,000*	442	89.1	54	10.9	-	-	-	-
**Marital Status**					0.573			
Never married	62	87.3	9	12.7	0.906	1.04	0.51	2.13
Divorced/widowed/separated	1798	87.8	250	12.2	0.292	1.18	0.87	1.62
Married *	334	85.9	55	14.1	-	-	-	-
**Current Job Status**					0.152			
Unemployed	436	85.0	77	15.0	0.043	1.40	1.01	1.93
Housewives	679	86.9	102	13.1	0.258	1.19	0.88	1.60
Retired	328	89.1	40	10.9	0.855	0.96	0.65	1.43
Employed*	751	88.8	95	11.2	-	-	-	-
**BMI (kg/m**^**2**^**)**					0.440			
< 25.0*	676	86.8	103	13.2	-	-	-	-
≥ 25.0	915	88.5	119	11.5	0.271	0.85	0.64	1.13
≥ 30.0	603	86.8	92	13.2	0.993	1.00	0.74	1.35
**Abdominal Obesity**								
No*	475	88.0	65	12.0	-	-	-	-
Yes	1719	87.3	249	12.7	0.70	1.06	0.79	1.42
**Sr. Cholesterol level (mmol/l)**								
<5.2*	1545	87.4	222	12.6	-	-	-	-
≥ 5.2	649	87.6	92	12.4	0.919	0.99	0.76	1.28
**HbA1c level (%)**								
≤8.5*	1376	89.5	161	10.5	-	-	-	-
>8.5	817	84.2	153	15.8	0.000	1.60	1.26	2.03
**Duration of Diabetes (years)**								
< 2	182	88.3	24	11.7	-	-	-	-
≥ 2*	2012	87.4	290	12.6	0.694	0.915	0.59	1.43
**Co-morbidity**								
No*	477	90.0	53	10.0	-	-	-	-
Yes	1717	86.8	261	13.2	0.049	1.37	1.00	1.87
**Psychiatric illness in family**								
No*	2179	87.7	305	12.3	-	-	-	-
Yes	15	62.5	9	37.5	0.001	4.28	1.86	9.88
**Diabetes in family**								
No*	594	89.9	67	10.1	-	-	-	-
Yes	1600	86.6	247	13.4	0.031	1.37	1.04	1.82
**Life events within the past 6 months**								
No*	1227	89.4	146	10.6	-	-	-	-
Yes	967	85.2	168	14.8	0.002	1.46	1.15	1.85
**Smoking status**					0.836			
Non smoker*	1824	87.3	265	12.7	-	-	-	-
Former smoker	142	88.8	18	11.2	0.743	0.94	0.63	1.39
Current smoker	228	88.0	31	12.0	0.598	0.87	0.53	1.45
**Current drinker**								
No*	2119	87.8	295	12.2	-	-	-	-
Yes	75	79.8	19	20.2	0.023	1.82	1.08	3.05
**Leisure-time physical activity level**								
Active*	1112	87.1	164	12.9	-	-	-	-
Inactive	1082	87.8	150	12.2	0.608	0.94	0.74	1.19

_c_OR –Crude odds ratio.

*- Reference group.

^†^ - Scores of 0–14 (normal).

‡ − Scores of ≥ 15 (mild, moderate, severe, extremely severe).

Variables with a p value of < 0.25 in the bivariate analyses and thought to be important risk factors of depression, anxiety and stress were entered into the multivariate model. The forward likelihood ratio (LR) method was used to predict the associated variables for depression, anxiety and stress symptoms in three separate models. The presence of interaction between the explanatory variables was assessed prior to determining the final model.

In the final model six variables i.e., sex, ethnicity, marital status, duration of diabetes, psychiatric illness in the family and alcohol consumption were found to be predictors of depression (Table 5). The strongest predictor was psychiatric illness history with an adjusted odds ratio (aOR) of 2.8 times followed by marital status (aOR 2.5-2.1) and current alcohol consumption (aOR 1.8). Individuals with diabetes of less than two years duration were 1.6 times more likely to have depressive symptoms than individuals with diabetes of longer duration while females and Asian Indians were 1.4 times more likely to have depressive symptoms compared to males and Malay diabetic individuals.

**Table 5:** Multiple logistic regression model predicting depression, anxiety and stress symptoms among type II diabetic outpatients

**Variable**	**Categories**	**B**	**S.E.**	**Wald**	**p-value**	**aOR**	**95% CI**	
							**Lower**	**Upper**
**Depression**								
**Constant**		−2.194	0.226	94.045	0.000			
**Ethnicity**				6.539	0.038			
	Malay*	-	-	-	-	-	-	-
	Chinese	−0.002	0.185	0.000	0.993	1.00	0.70	1.44
	Asian Indian	0.337	0.142	5.619	0.018	1.40	1.06	1.85
**Sex**	Male*	-	-	-	-	-	-	-
	Female	0.350	0.145	5.828	0.016	1.42	1.068	1.89
**Marital Status**				29.490	0.000			
	Never married	0.931	0.300	9.656	0.002	2.54	1.41	4.57
	Divorced/widowed/separated	0.735	0.153	22.935	0.000	2.09	1.54	2.82
	Married*	-	-	-	-	-	-	-
**Psychiatric illness in family**	No*	-	-	-	-	-	-	-
	Yes	1.014	0.469	4.685	0.030	2.76	1.10	6.91
**Duration of diabetes (years)**	< 2	0.452	0.205	4.840	0.028	1.57	1.05	2.35
	≥ 2*	-	-	-	-	-	-	-
**Current drinker**	No*	-	-	-	-	-	-	-
	Yes	0.610	0.312	3.823	0.051	1.84	1.00	3.39
**Anxiety**								
**Constant**		−1.474	0.293	25.217	0.000	0.229		
**Age Group (yrs)**				11.180	0.048			
	30 – 39*	-	-	-	-	-	-	-
	40 - 49	−0.530	0.201	6.955	0.008	0.59	0.40	0.87
	50 - 59	−0.402	0.188	4.559	0.033	0.67	0.46	0.97
	60 – 69	−0.468	0.202	5.378	0.020	0.63	0.42	0.93
	70 - 79	−0.138	0.238	0.337	0.562	0.87	0.55	1.39
	≥ 80	−0.400	0.416	0.922	0.337	0.67	0.30	1.52
**Current Job Status**				17.848	0.001			
	Retired	0.201	0.160	1.579	0.209	1.22	0.89	1.67
	Unemployed	0.220	0.140	2.448	0.118	1.25	0.95	1.64
	Housewives	0.474	0.158	9.020	0.003	1.61	1.18	2.19
	Employed*	-	-	-	-	-	-	-
**HbA1c level (%)**	≤8.5*	-	-	-	-	-	-	-
	>8.5	0.298	0.091	10.649	0.001	1.35	1.13	1.61
**Psychiatric illness in family**	No*	-	-	-	-	-	-	-
	Yes	0.876	0.419	4.368	0.037	2.40	1.06	5.46
**Life events**	No*	-	-	-	-	-	-	-
	Yes	0.324	0.089	13.241	0.000	1.38	1.16	1.65
**Physical Activity Status**	Inactive	0.304	0.090	11.542	0.001	1.36	1.13	1.62
	Active*	-	-	-	-	-	-	-
**Stress**								
**Constant**		−2.832	0.193	215.60	0.000	0.059		
**Sex**	Male*	-	-	-	-	-	-	-
	Female	0.312	0.134	5.397	0.020	1.37	1.05	1.78
**HbA1c level (%)**	≤8.5*	-	-	-	-	-	-	-
	>8.5	0.493	0.123	16.119	0.000	1.64	1.29	2.08
**Co-morbidity**	No*	-	-	-	-	-	-	-
	Yes	0.345	0.161	4.561	0.033	1.41	1.029	1.94
**Psychiatric illness in family**	No*	-	-	-	-	-	-	-
	Yes	1.435	.434	10.931	0.001	4.20	1.79	9.83
**Life events**	No*	-	-	-	-	-	-	-
	Yes	0.292	0.123	5.592	0.018	1.34	1.05	1.71
**Current drinker**	No*	-	-	-	-	-	-	-
	Yes	0.775	0.282	7.580	0.006	2.17	1.25	3.77

aOR –adjusted odds ratio.

*- Reference group.

In terms of anxiety (Table 5), age group, job status, HbA1c, family history of psychiatric illness, life events and leisure-time physical activity remained significant in the final model, while for stress five variables namely sex, HbA1c, co-morbidity, psychiatric illness in the family, life events and alcohol consumption were found to be significant contributors. Individuals with a family history of psychiatric illness were 2.4 times more likely to report anxiety. Anxiety was 1.4 times more likely in respondents experiencing life events, those physically inactive and with an HbA1c level of >8.5%. All diabetics who were not gainfully employed were 1.2-1.6 times more likely of experiencing anxiety.

Psychiatric illness in the family was the strongest predictor of stress having aOR of 4.2 followed by current alcohol drinkers with an aOR of 2.2. Having a highly undesirable level of HbA1c was associated with at least 1.6 times the odds of stress. Female diabetics and those with co-morbidity were 1.4 times more likely to report stress after controlling for confounders.

There was also significant correlation (p < 0.01) between depression, anxiety and stress symptoms (r = 0.360 for depression and anxiety, r =0.547 for depression and stress and r = 0.504 for anxiety and stress).

## Discussion

### Depression, anxiety and stress

This study showed that the prevalence of depression, anxiety and stress symptoms were 11.5%, 30.5% and 12.5% respectively among Type II Diabetic outpatients in the Klang Valley, Malaysia. The prevalence of anxiety in this study was almost three fold more than that of depression and this is in keeping with current literature in which anxiety rates are frequently higher than depression [39-42].

Our findings concur with other chronic disease models such as chronic obstructive pulmonary disease (COPD), whereby a study by Dahlen & Janson found that 12% and 37% of respondents with asthma and COPD had probable depression and anxiety respectively [43].

The depressive symptom rates we found are also comparable to studies in rural America (15.8%) [44], the UAE (12.5%) [45] and Germany (10.2%) [11]. Conversely, several studies among diabetic patients had found higher rates than our study [39,40,46]. A study in Qatar using the same instrument as ours, i.e., DASS 21 found more than half of the diabetics have depressive, anxiety and stress symptoms [47]. The sex specific depression rates in our study were found to be within the estimated range in the general population. The differences in the rates of depression, anxiety and stress symptoms between our study and others may be attributed to differences in the screening or diagnostic instruments used, the socio-cultural differences of different populations and also the sample size of the subjects. In particular, the study from Qatar had a smaller sample size of 1788.

Our study revealed that sex, ethnicity, marital status, duration of diabetes, psychiatric illness in the family and alcohol consumption were predictors of depression. These findings are consistent with other studies which also found sex [9,39,40,48,49], ethnic minority groups [50] and duration of diabetes [47] significantly associated with depression among diabetics. It is not surprising that females have a higher prevalence and risk of depression compared to males. Many factors have been implicated for this gender difference including socio-cultural and biological factors [51]. As for ethnicity, minority ethnic groups have been found to have higher depression rates as quoted in other studies [52,53]. It could be theorized that Asian Indians being the minority are more likely than Malays who form the majority to be exposed to a gamut of psychosocial stressors such as enhanced socioeconomic constraints, poor education and perceived discrimination. Consequently, these issues might augment distress thereby increasing the levels of depression among this group of minority. Similar findings and explanations were cited in a community based cross-sectional study in the United States comparing depression and anxiety in respondents having insomnia and respondents without among the majority Caucasians and minority African American. In that study, Taylor et al. [54] established that African Americans were more likely to have clinically significant depression and anxiety. This was attributable to the plausible exposure to greater array of stressors in the form of discrimination, socioeconomic adversities and enhanced caregiver burden for African Americans as compared to Caucasians.

Generally, it is recognized that being married is associated with less psychiatric morbidity including depression [55-57]. Our finding of previously married and never married individuals being associated with depressive symptoms was compatible with a study by Aqbir et al. [48]. It is very likely that having a partner or spouse in a stable marriage offers emotional stability as well as shared burden in coping with challenges.

Incidentally a duration of less than 2 years of having diabetes was a predictor of depressive symptoms in this study. This may be attributed to inadequate or inefficient coping skills of managing diabetes by the respondents within the relatively short period since diagnosis. A related study conducted in Bahrain affirmed an association between duration of diabetes and depressive symptoms [58].

Additionally, this study elucidated the strongest predictor of depressive symptoms was having a family history or family member with psychiatric illness. It is recognized that mental illnesses in general tend to run within families [59]. In addition, in an Asian society such as ours, the responsibility of the family in caring for an ill member whether physically, emotionally or financially is very much entrenched in the culture. Hence, the caregiver’s burden is occasionally translated into physical and mental health adversities such as depression [60,61].

In terms of alcohol consumption, our study showed that current drinking was an independent risk factor for depression albeit marginally. Several studies have established such an association [41,42,62].

For anxiety, our study demonstrated that having a family history of psychiatric illness was the strongest predictor. The finding is not surprising for the same reasons mentioned as for depression. That an elevated HBA1c level was an independent risk factor of anxiety in our study is also echoed by a similar association between blood glucose level and anxiety in a Mexican population [40].

With reference to current job status, this study noted that housewives and those unemployed were at risk of reporting anxiety symptoms than those who were gainfully employed. It is likely that unemployed persons lack feelings of stability and this could contribute to feelings of anxiety. However, further research is needed to show the reasons for our finding.

In this present era of modernization, anxiety has considerable influence upon the quality of life. Balancing work, family and leisure time is a challenge for both working men and women nowadays. If there was a life event preceding which could upset this delicate balance, there would definitely be an increased predilection to develop anxiety in the affected individuals. Our findings concur with several other studies conducted in this area [63,64].

Physical activity has been shown to promote feelings of well being. Our findings harmonize with other studies showing association between physical inactivity and anxiety symptoms [39,65]. Furthermore, regular physical activity has revealed a reduction of anxiety symptoms in those who already suffer from this disorder [66].

Our data inferred that having a family member with psychiatric illness was the strongest predictor of stress. Once again caring for a family member who has a chronic illness like mental disorders is indeed a challenging task that could increase the possibilities of adversities [60,61].

Pertaining to alcohol consumption, current drinkers were strongly associated with stress symptoms. Studies have shown that drinking is used as means of coping with life’s stresses [67]. Child’s et al. have suggested a bidirectional relationship between alcohol use and stress [68].

Experiencing life events is inherently stressful and thus can complicate any stress level already existing in an individual [69]. Lyod et al. [70] have noted that recent severe stressors were associated with poorer glycemic control while another study found a significant correlation between stress and HbA1c [71]. Hence, our findings of life events and elevated HbA1c levels being independently associated with stress symptoms are consistent. Females were found to be at risk of stress in this study. The finding is in accordance with current literature on gender differences and stress symptoms [72-74]. We did not find any association between other socio-demographic, lifestyle and clinical factors with stress symptoms.

The strength of our study lies in its large sample size and the sampling method. However, there are also limitations which need to be considered. Firstly, this being a cross sectional study does not allow for cause and effect relationships to be studied. Secondly, the DASS 21 questionnaire is only a screening tool and not diagnostic of specific psychiatric disorders. There is also the possibility of recall biases from respondents, however this was minimized by limiting the recall period to 1 week prior to the interview using DASS 21. Information such as age, past medical history and types of medication was also verified with medical records where applicable. A point to note is that the low prevalence of family history of psychiatric illness elicited from the study could be a result of under reporting as psychiatric illness is still viewed with suspicion and stigma in our society.

As part of a long term and holistic diabetes care management, we recommend that screening for depression, anxiety and stress symptoms be conducted at regular intervals for Type II diabetics with vulnerable characteristics as mentioned above. Policies need to be in place along with appropriate intervention so that these vulnerable individuals receive optimal and timely mental health care, thus translating into better overall health outcomes.

## Conclusions

The study showed that while the prevalence of depression and stress symptoms was just over 10%, almost a third were classified as anxious. As these symptoms are highly correlated, they should be considered together when managing diabetic patients. Our findings could help primary care physicians identify high risk diabetics for screening of mental disorders. A family history of psychiatric illness was found to be a common predictor for all three symptoms. Females, current alcohol drinkers, experiencing recent life events and poor glycemic control were the other common predictors of at least two of these symptoms.

## Competing interests

The authors declare that they have no competing interests.

## Authors’ contributions

GK was involved in the conception and design of the project, management of data collection/entry, cleaning, statistical analyses and manuscript writing/revising. TGH assisted in the project management, data collection and manuscript writing/revising. SA was involved in the design of the project, assisted in coordination of data collection and manuscript writing/revising. ASK assisted in the project design, conducted the data collection and manuscript writing and revising. KC assisted in the design of the project, data management and statistical analyses. All authors read and approved the final manuscript.

## Pre-publication history

The pre-publication history for this paper can be accessed here:

http://www.biomedcentral.com/1471-2296/14/69/prepub

## Supplementary Material

Additional file 1 DAS S 21. Click here for file
