# Supplementary material for: Depression, anxiety and stress symptoms among diabetics in Malaysia: a cross sectional study in an urban primary care setting
Source: BMC Fam Pract. 2013 May 27;14:69. doi: 10.1186/1471-2296-14-69 (PMC3669622; doi:10.1186/1471-2296-14-69)
Supplement: Additional file 1 — DAS S 21. [file 1471-2296-14-69-S1.doc]

APPENDIX

| DAS S 21 | | |
| --- | --- | --- |
| Please read each statement and circle a number 0, 1, 2 or 3 which indicates how much the statement applied to you *over the past week*. There are no right or wrong answers. Do not spend too much time on any statement. | | |
| *The rating scale is as follows:*  0 Did not apply to me at all  1 Applied to me to some degree, or some of the time  2 Applied to me to a considerable degree, or a good part of time  3 Applied to me very much, or most of the time | | |
| 1 | I found it hard to wind down | 0 1 2 3 |
| 2 | I was aware of dryness of my mouth | 0 1 2 3 |
| 3 | I couldn't seem to experience any positive feeling at all | 0 1 2 3 |
| 4 | I experienced breathing difficulty (eg, excessively rapid breathing, breathlessness in the absence of physical exertion) | 0 1 2 3 |
| 5 | I found it difficult to work up the initiative to do things | 0 1 2 3 |
| 6 | I tended to over-react to situations | 0 1 2 3 |
| 7 | I experienced trembling (eg, in the hands) | 0 1 2 3 |
| 8 | I felt that I was using a lot of nervous energy | 0 1 2 3 |
| 9 | I was worried about situations in which I might panic and make a fool of myself | 0 1 2 3 |
| 10 | I felt that I had nothing to look forward to | 0 1 2 3 |
| 11 | I found myself getting agitated | 0 1 2 3 |
| 12 | I found it difficult to relax | 0 1 2 3 |
| 13 | I felt down-hearted and blue | 0 1 2 3 |
| 14 | I was intolerant of anything that kept me from getting on with what I was doing | 0 1 2 3 |
| 15 | I felt I was close to panic | 0 1 2 3 |
| 16 | I was unable to become enthusiastic about anything | 0 1 2 3 |
| 17 | I felt I wasn't worth much as a person | 0 1 2 3 |
| 18 | I felt that I was rather touchy | 0 1 2 3 |
| 19 | I was aware of the action of my heart in the absence of physical exertion (eg, sense of heart rate increase, heart missing a beat) | 0 1 2 3 |
| 20 | I felt scared without any good reason | 0 1 2 3 |
| 21 | I felt that life was meaningless | 0 1 2 3 |
